# Microalgae Peptide-Stabilized Gold Nanoparticles as a Versatile Material for Biomedical Applications

**DOI:** 10.3390/life12060831

**Published:** 2022-06-02

**Authors:** Marielys Torres-Díaz, Caren Abreu-Takemura, Liz M. Díaz-Vázquez

**Affiliations:** 1 Department of Chemistry, University of Puerto Rico-Río Piedras Campus, San Juan 00925, Puerto Rico; marielys.torres2@upr.edu; 2 Department of Biology, University of Puerto Rico-Mayagüez Campus, Mayagüez 00680, Puerto Rico; caren.abreu@upr.edu

**Keywords:** AuNPs-Colloidal Stability, *Chlorella* peptide coating, ecotoxicity of AuNPs, microalgae drug delivery systems

## Abstract

Microalgae peptides have many medical and industrial applications due to their functional properties. However, the rapid degradation of peptides not naturally present in biological samples represents a challenge. A strategy to increase microalgae peptide stability in biological samples is to use carriers to protect the active peptide and regulate its release. This study explores the use of gold nanoparticles (AuNPs) as carriers of the *Chlorella* microalgae peptide (VECYGPNRPQF). The potential of these peptide biomolecules as stabilizing agents to improve the colloidal stability of AuNPs in physiological environments is also discussed. Spectroscopic (UV-VIS, DLS) and Microscopic (TEM) analyses confirmed that the employed modification method produced spherical AuNPs by an average 15 nm diameter. Successful peptide capping of AuNPs was confirmed with TEM images and FTIR spectroscopy. The stability of the microalgae peptide increased when immobilized into the AuNPs surface, as confirmed by the observed thermal shifts in DSC and high zeta-potential values in the colloidal solution. By optimizing the synthesis of AuNPs and tracking the conferred chemical properties as AuNPs were modified with the peptide via various alternative methods, the synthesis of an effective peptide-based coating system for AuNPs and drug carriers was achieved. The microalgae peptide AuNPs showed lower ecotoxicity and better viability than the regular AuNPs.

## 1. Introduction

Microalgae are photosynthetic organisms extensively studied as a source of biofuel, food, nutraceuticals, and supplements [1,2]. These microorganisms produce a variety of bioactive compounds with potential anti-microbial, anti-cancerous, anti-inflammatory, and health-promoting effects. The biomedical properties of microalgae are primarily associated with their phytochemical components, especially the secondary metabolites, which are sources of value-added bioactive compounds [3]. Microalgae bioactive peptides are involved in the defense response, cellular signaling, and development regulation [4]. Peptides derived from microalgae are of great interest due to their functional properties, such as solubility, emulsifying, and foaming properties, benefiting medical and industrial applications [5]. In addition, the fact that microalgae peptides can be obtained in in vivo or in vitro approaches using different techniques, such as enzymatic hydrolysis of extracted proteins or by proteins from an ingested algae biomass, has facilitated their production and availability for pharmaceutical applications [5,6,7]. Furthermore, various synthetic strategies have been developed over the years to modulate the conformational flexibility and the peptide character of peptidomimetic compounds [8]. In the last decade, microalgae peptides from *Chlorella* sp., *Dunaliella* sp., and *Pavlova* sp. have been investigated because of their high potential for use in pharmaceuticals due to their cancer-inhibiting or immunomodulating properties [9,10,11,12].

*Chlorella vulgaris* is a green unicellular organism and one of the most commercially used microalgae. This is primarily due to its high production rates, uncomplicated cultivation requirements, and its status as FDA-approved for human consumption [1]. This microalga has a unique and diverse chemical composition of functional macro- and micro-nutrients, including proteins, peptides, omega-3 polyunsaturated fatty acids, polysaccharides, vitamins, and minerals, with biological and pharmacological properties important for human health [13,14,15]. The bioactive *Chlorella vulgaris* peptides have been shown to possess different biological functionalities, such as antioxidant, anticancer, antihypertensive, and antimicrobial activities, with beneficial health effects and potential therapeutic applications [16]. Research evidence has shown that a peptide extracted from *Chlorella vulgaris*, with sequence VECYGPNRPQF, contains strong antioxidant properties and anti-cancer potential, although very little is known about the specific mechanisms of action [12]. However, the rapid degradation of peptides that are not naturally present in human blood is a challenge that needs to be addressed. Maximizing the effects of biomedical interest microalgae peptides like VECYGPNRPQF relies heavily on their stability with external factors, such as pH and temperature, in biological systems [17]. A strategy to increase microalgae peptide stability in biological and environmental samples is to use carriers to protect the active peptide and regulate its release at target areas or cells.

Gold nanoparticles (AuNPs) have shown promise as effective drug carriers for targeted treatment in the biomedical field. Advantages of using AuNPs can be firstly reflected in their low toxicity. Intravenous administration in various dosages of spherical AuNPs ranging from 10–18 nm in diameter has shown no morphological changes, renal toxicity, or hematological interferences in mice [18]. The same study showed that these AuNPs tended to accumulate in specific organs, such as the kidney, spleen, and liver, without a trace of tissue damage. Because AuNPs seem to be readily incorporated into organs, it supports the possibility that non-malignant targeted treatment can be made to specific parts of the body with proper functionalization. However, poor colloidal stability of gold nanoparticles (AuNPs) in physiological environments remains one of the significant limitations contributing to their difficult translation from bench to clinic studied for biomedical purposes. To tackle this limitation, scientists are taking advantage of AuNPs’ capacity to be modified, especially with agents with thiols in them. AuNPs’ surfaces could be modified to tailored properties conferred by capping/protecting agents, including N-, P-, COOH-, and SH-containing molecules and polymers; chelating agents; and amines, among other chemical functionalities [19]. These modifications provide metal nanoparticles with specific properties, such as size, shape, dispersion, chemical miscibility, and activity, contributing to avoiding their aggregation and oxidation over time and improving their stability [20,21,22].

Due to gold-thiol affinity, many studies have focused on the self-assembling modifications of AuNPs, such as conjugation with L-cysteine and PEG-thiols for specific applications. This is how a PEG-thiol modified AuNPs served peptide linking for better vaccine delivery in cancer immunotherapy [23]. A critical aspect noted in that study was that it interestingly facilitated the endocytosis of AuNPs, specifically by dendritic cells. This provides valuable insights, because even when AuNPs themselves can be absorbed by cells, as shown by the mice, it exemplifies that different coatings of these nanoparticles open a wide variety of nanoparticle conjugation with macromolecules that may favor the same endocytosis effect. The antibacterial activity of bioactive molecules in gold nano-carriers was studied as an infection treatment in mice models [24]. This study demonstrated very efficient antibacterial activity and good biocompatibility in this in vivo experiment.

Herein, we proposed the use of AuNPs as carriers of the microalgae peptides while exploring the potential of this modification as a stabilizing agent to improve the colloidal stability of gold nanoparticles (AuNPs) in physiological environments. The peptide’s 11-unit sequence contains a cysteine from which its thiol group can enable direct and robust interactions with AuNPs for a potential drug-carrying system. Because the accumulation, endocytosis, and transport of AuNPs in the body rely on their size and morphology, citrate reductions were the synthesis of choice to regulate the homogeneity of these two characteristics.

*Aliivibrio fischeri* (also known as *Vibrio fischeri*) is a marine bioluminescent bacterium, which was applied as a model microorganism to monitor the toxicity and bioavailability of the microalgae peptide gold nanoparticles. *A. fischeri* has been used to study the toxicity of metallic nanoparticles such as ZnO, CuO, and TiO_2_ [25]. However, these studies are limited to a Flash assay exposition of the bacteria. A more complex approach to evaluating the chronic toxicity of the peptide-modified gold nanoparticles (pep-AuNPs) compared to citrate-stabilized AuNPs (cit-AuNPs) is proposed as an ecotoxicity assay. The interaction between algae and marine microbiota is not fully understood, but could affect their metabolism and behavior [26]. The development of new nanomaterial with enhanced properties containing bioactive compounds from algae could impact marine ecosystems if these materials are released into the environment.

## 2. Materials and Methods

### 2.1. Synthesis of Gold Nanoparticles and Peptide Modification

**Figure 1:**
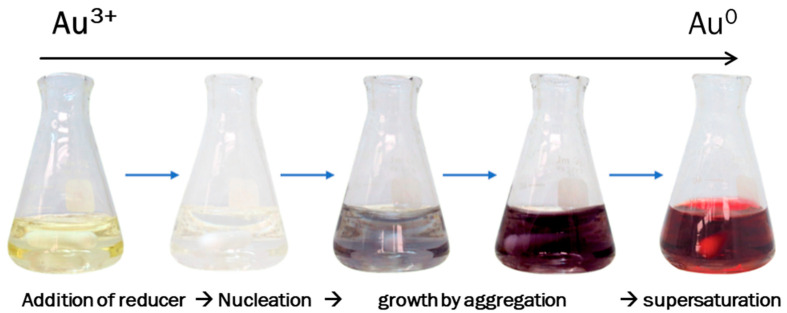
Progress of the gold nanoparticles synthesis reaction. The yellow-colored solution corresponds to the Gold (III) ions in water. When the citrate solution is added, the mixture turns clear, and further color changes occur until the mixture turns an intense red color at a supersaturation stage.

Gold nanoparticles (cit-AuNPs) were synthesized using the citrate-reduction method [27]. The gold solution was prepared by dissolving tetrachloroauric acid (HAuCl_4_·XH_2_O from Sigma-Aldrich, St. Lois, MO, USA) in nanopure water for a final concentration of 1 mM, while the sodium citrate (Sigma-Aldrich) was prepared at a concentration of 1% (*w*/*v*) in nanopure water. An aliquot of 20 mL of gold solution was placed in a flask and heated with continuous stirring until boiling. Eventually, 5 mL of citrate solution was added to the boiling gold solution. The mixture was stirred and heated until a color change was observed from yellow to red (Figure 1). For the peptide modification (pep-AuNPs), a peptide solution was prepared with a 1 mg/mL concentration in nanopure water. The peptide (VECYGPNRPQF) was ordered as a custom synthesis from Biomatik©. An aliquot of 100 μL of the peptide solution was added dropwise to 5 mL of gold nanoparticles [28]. The mixture was constantly stirred at room temperature for 2 h. Finally, the nanoparticles were centrifuged and washed with nanopure water.

### 2.2. Characterization of Gold Nanoparticles

#### 2.2.1. UV-VIS Spectroscopy

The absorption spectrum (250–750 nm) was obtained for each sample to determine the absorption maximum and its plasmon resonance behavior related to the shape and size of the nanoparticles. The measurements were performed in a Thermo Scientific Multiskan Go Spectrophotometer with the cuvette mode.

#### 2.2.2. Dynamic Light Scattering and Z-Potential

The hydrodynamic radius and the Z-potential of AuNPs in solution were physically characterized using a Malvern ZetaSizer Nano Series (4 mW, 632.8 nm laser) in low volume disposable DLS cuvettes at 25 °C, equipped at a scattering angle of 90°. Analyses were performed in water (viscosity: 0.8872 Cp, refractive index: 1.33). The size measurements were averaged from at least three repeated measurements. 

#### 2.2.3. FTIR Spectroscopy

A small volume of the gold nanoparticles suspension was lyophilized to remove the water from the sample. The dry powder was mixed with KCl (Sigma-Aldrich) and homogenized with a mortar and pestle. A very fine powder was used for the measurement. The analysis was performed using a Shimadzu Corp IRAffinity-1S FTIR with a frequency range of 4000–400 cm^−1^ with 100 scans and a resolution of 4 cm^−1^.

#### 2.2.4. Transmission Electron Microscopy

Bright Field TEM images of the gold nanoparticles were obtained with a FEI F20 S/TEM (200 kV field emission, 1 Å monochromator) at 71,000× magnification. The images were analyzed with the program ImageJ to determine the size distribution of the nanoparticles (101 nanoparticles per sample). Microsoft Excel Software was used to create the histograms and the Gaussian fit. A total of 1000 values of nanoparticles diameter (nm) were generated considering the same mean and standard deviation of the experimental data set but a normal distribution. Then, the data were sorted within the limits of the bins used for the histograms and graphed as a combined figure with both data sets.

#### 2.2.5. Agarose Electrophoresis

The stability of gold nanoparticles and their relative charge was studied using the difference in migration of the different AuNPs through an electrophoresis gel [29,30,31]. The running buffer used was Tris-borate-EDTA 1X (TBE 1X). The agarose gel was prepared at a 1% (*w*/*v*) concentration in TBE 1X with an 8-well comb. The loading buffer for the samples was prepared by mixing equal parts (1:1) of glycerol and SDS 10% solution in water. A volume of 100 μL of nanoparticles suspension was mixed with 12.5 μL of loading buffer. Each well was filled with 40 μL of the sample mixture, and the run was performed at 100 V for 30 min. All the reagents used were purchased from Sigma-Aldrich.

#### 2.2.6. Differential Scanning Calorimetry

Thermal analyses of lyophilized cit-AuNPs and pep-AuNPs were performed on a Differential Scanning Calorimeter METTLER TOLEDO DSC822e. Compressed nitrogen gas was used through a Hammond Drierite gas purifier stock#27068 as the purging agent during all runs. Samples were placed in a 40 μL aluminum pan. The citrate samples (pure sodium citrate and cit-AuNPs) were subjected to heating from 25–450 °C, at a rate of 10 °C/min, while the peptide samples (pure peptide and pep-AuNPs) were heated from −10–450 °C, at the same heating ramp of 10 °C/min.

#### 2.2.7. ABTS Antioxidant Capacity

An ABTS (Sigma-Aldrich) 7 mM solution and a sodium persulfate (Sigma-Aldrich) 2.4 mM solution were prepared in nanopure water [32,33,34]. Equal parts of each solution were mixed into an amber container and incubated in a dark place for 12–16 h to activate the ABTS radical. The mixture was diluted in ethanol until the absorbance measurement at 734 nm was approximately 0.85. To determine the IC50 concentration, serial dilutions (6–8) were prepared for cit-AuNPs and pep-AuNPs. A 96-well microplate was used to measure the scavenging capacity of the samples in triplicate. To prepare the plate, 25 μL of the sample and 175 μL of diluted ABTS were added to each well. This same proportion was used for the control samples. The plate was incubated in a dark place for 5 min before measuring the absorbance at 734 nm using the Thermo Scientific Multiskan Go Spectrophotometer with the plate reader mode. The scavenging capacity was calculated with the formula:

$$\mathrm{scavenging}\;\%=\frac{\left[Abs\;\left(control\right)-Abs\;\left(sample\right)\right]}{Abs\;\left(control\right)}\;\times \;100$$

Microsoft Excel Software was used to calculate de IC50 for cit-AuNPs and pep-AuNPs. The scavenging % was plotted per each concentration used, a linear regression was applied to the data set, and the equation was obtained. The concentration needed to deactivate 50% of the free radicals was calculated with this information.

### 2.3. Ecotoxicity Assay with Aliivibrio Fischeri

#### 2.3.1. Bacterial Culture

The bacterial strain used was the wild type *Aliivibrio fischeri* ES114. A frozen glycerol stock of ES114 was stored at −80 °C. The first step in the bacterial culture was the agar plate streaking using LBS media. LBS media contained, per liter of distilled water, 10 g of Tryptone, 5 g of Yeast Extract, 20 g of NaCl, 15 g of agar, and 50 mM Tris (pH 7.50) [35]. The agar streaking was incubated at 24 °C for 12 h. Single colonies were recovered from the plate and inoculated in liquid LBS media (1 colony per 3 mL of LBS). This liquid culture was incubated for at least 7 h at 24 °C and 200 rpm. A second inoculation using 5 μL of seed culture for 5 mL of liquid LBS was prepared and incubated for 12 h at 24 °C and 200 rpm.

#### 2.3.2. Growth Pattern and Bioluminescence Measurements

Bacterial cultures were exposed to cit-AuNPs and pep-AuNPs to study their growth pattern and bioluminescence emission. The effects of the peptide used for the nanoparticle’s modification were also evaluated at the same final concentration used in the reaction. The experiment was performed using a sterile Corning 96-well black plate with a clear bottom and lid. Two different culture media were used: SWTO, a rich medium (contains 5 g Tryptone, 3 g Yeast extract, 20 g NaCl, 700 mL Instant Ocean 36 ppt, and 3 mL glycerol; additional NaCl is used to adjust the final salinity to 43 ppt and distilled water to adjust the volume to 1 L), and FMM, a minimal medium (contains 950 mL distilled water, 50 mL 1 M Tris (pH 7.5), 378 μL 1 M sodium phosphate (pH 7.5), 0.59 g NH_4_Cl, 0.83 g KCl, 19.5 g NaCl, 3 mg FeSO_4_·7H_2_O, 13.6 g MgSO_4_·7H_2_O, 1.62 g CaCl_2_·2H_2_O, and 3 mL of glycerol) [35]. All the reagents used for the culture media were purchased from Thermo Fisher. The bacteria grown in LBS were inoculated in each of the media with a proportion of 1:1000. Each of the wells in the plate was filled with a maximum volume of 200 μL. The plate was prepared with nine replicas per sample, which included: Positive control in both media (C+ ES114); Negative control of sterile media (C−); and the groups exposed to cit-AuNPs, pep-AuNPs, and the peptides in both media. Samples prepared with nanoparticles and peptides had 195 μL of the inoculated media and 5 μL of the nanoparticles, while the controls were filled with 200 μL of the corresponding sample. A TECAN Infinite 200Pro was used with the software i-control to measure absorbance at 600 nm (Optical density: OD 600) and luminescence emission every 30 min for 10 h. The instrument was configured to maintain a temperature of 24 °C and perform orbital shaking at 200 rpm before each measurement. The data were analyzed with Microsoft Excel and RStudio.

## 3. Results and Discussion

**Figure 2:**
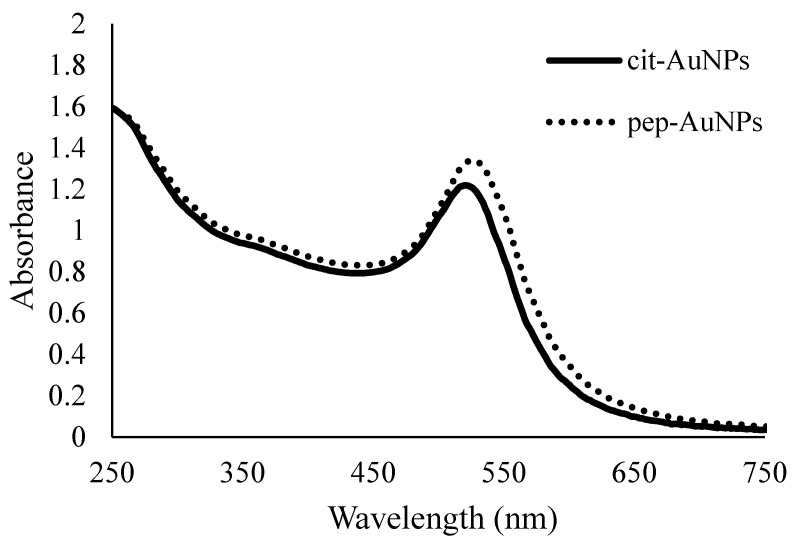
UV-VIS absorption spectra of the citrate-stabilized gold nanoparticles (cit-AuNPs) and the peptide-functionalized gold nanoparticles (pep-AuNPs). The corresponding maximum absorption wavelength for the cit-AuNPs was 522 nm, and for the pep-AuNPs, it was 526 nm.

The proposed method for the surface functionalization of AuNPs with *Chlorella* microalgae peptide (VECYGPNRPQF) was successful, as confirmed by the characterization information obtained with different analytical techniques. AuNPs have optical properties that are characteristic of their size and morphology. UV-VIS Spectroscopy was used to determine the maximum wavelength of absorption (λ_max_) of the AuNPs, since this value is related to the surface resonance plasmon, which is dictated by the size, morphology, and surface chemical interactions of the metallic nanoparticles [36]. The absorbance spectra of the synthesized AuNPs, as shown in Figure 2, demonstrate the λ_max_ of cit-AuNPs to be 522 nm and of the pep-AuNPs to be 526 nm. These values of λ_max_ have been reported to correspond to spherical AuNPs ranging between 20–30 nm in diameter [37]. However, slight shifts in these absorbance peaks may indicate differences in size and surface modification [36]. Therefore, complementary information obtained with other techniques was used to confirm sizes and surface properties.

**Figure 3:**
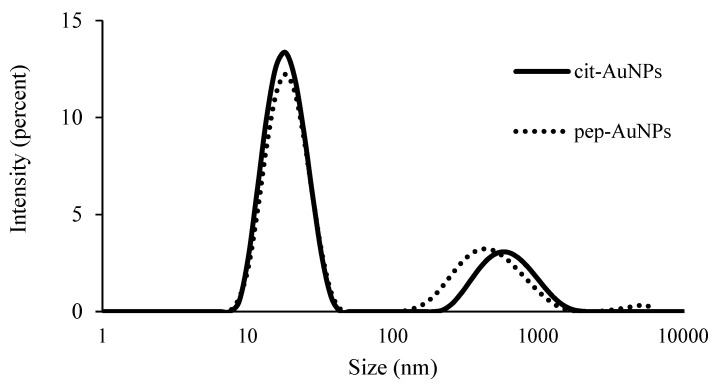
Size distribution of the gold nanoparticles determined by DLS measurements of intensity.

Dynamic Light Scattering (DLS) was performed to obtain information about the AuNPs’ particle size, polydispersity distribution, aggregation, and stability. Specific values of z-average, intensity percentages of peaks, polydispersity indexes, and Zeta-potential are summarized in Table 1. DLS diagram (Figure 3) shows the primary symmetric peak with high intensity at around 18 nm of diameter for the cit-AuNPs and approximately 20 nm for the pep-AuNPs. These values represent most of the particles in the solution. A smaller peak was observed for both samples at a higher value, indicating aggregation of the particles and not the presence of bigger particles. The PDI values support this at around 0.4 for both samples. PDI is used to estimate the uniformity of the particles in the solution, and the sample is considered monodispersed if the value is <0.1 [38]. On the other hand, if the value of PDI is >0.7, the sample is considered to have a broad distribution and, thus, is not suitable for DLS measurements [39]. Zeta-potential values describe the dispersion stability and provide information about the polymer’s affinity, in this case, a peptide used for the modification [22]. The surface modification of the nanoparticles was confirmed with the increased size of the pep-AuNPs regarding the peptide coating, and the decreased magnitude of the Zeta-potential indicates a less negative surface due to the shielding effect the neutral peptide strong interaction [22]. The measured values were −44.14 mV for the cit-AuNPs and −42.96 mV for the pep-AuNPs (Table 1). Generally, colloids with Zeta-potential values beyond ±30 mV are highly stable dispersions [40].

**Figure 4:**
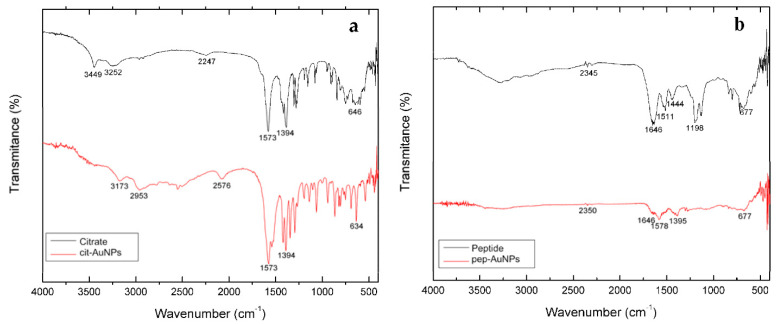
FTIR spectra of the nanoparticles and the stabilizing agents confirm the gold nanoparticles’ surface modification. (**a**) citrate and cit-AuNPs and (**b**) peptide and pep-AuNPs.

The Fourier Transformed-Infrared (FTIR) Spectroscopy analysis confirmed that cit-AuNPs were effectively capped by citrate (Figure 4a). The citrate spectrum showed distinctive bands, such as the stretching vibration of the OH group at 3449 cm^−1^ and symmetrical and antisymmetric C=O stretching of carboxylate ions at 1394 cm^−1^ and 1573 cm^−1^. These bands were also detected in the cit-AuNPs. However, in the cit-AuNP spectrum, the band at 3449 cm^−1^ was shifted into 3173 cm^−1^, indicating that the citrate anions are absorbed on AuNPs through central carboxylate groups [41]. The FTIR spectra for the peptide and the pep-AuNPs were compared (Figure 4b). The peptide modification of the nanoparticles was confirmed by the presence of amide I, amide II, and amide III bands, which correspond to the peptide, appearing in the pep-AuNPs spectrum [42]. The band characteristic of the amide I region (1600–1700 cm^−1^), 1646 cm^−1^, was detected in the pure peptide and pep-AuNPs samples, corresponding to the C=O stretch, which is weakly coupled with C-N stretch and N-H bending. The peak corresponding to the amide II region (1500–1600 cm^−1^) was detected at 1511 cm^−1^ for the peptide and displaced at 1578 cm^−1^ for the pep-AuNPs. This band corresponds to the C-N stretch strongly coupled with N-H bending. The constant shifting of bands in the obtained data suggests that the hydrogen binding pattern and, subsequently, backbone conformation of the peptide were affected by its attachment to the gold surface [43].

**Figure 5:**
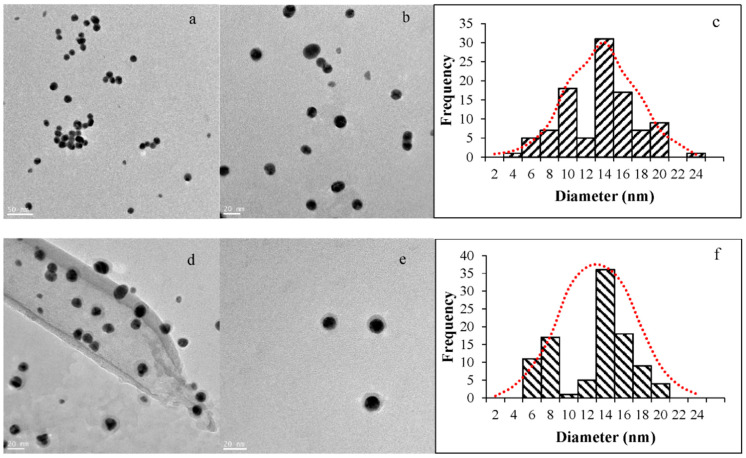
TEM images and histograms of the gold nanoparticles. (**a**,**b**) TEM images of cit-AuNPs; (**c**) Size distribution of the cit-AuNPs determined with ImageJ. (**d**,**e**) TEM images of pep-AuNPs; (**f**) Size distribution of the pep-AuNPs determined with ImageJ.

TEM images confirmed the spherical AuNPs expected from the citrate reduction method [44,45,46]. Figure 5a shows some aggregation of the cit-AuNPs, which is consistent with the DLS information. The size distribution of the cit-AuNPs is presented in Figure 5c, where the histogram is combined with a simulated normal distribution with the same average and standard deviation values as the experimental data to compare the tendency. As observed, the normal distribution is not a perfect fit to the bars in the histogram and is not presented as a smooth line. The experimental data show different higher frequency regions consistent with the polydispersity predicted by the DLS analysis. TEM images from the pep-AuNPs are presented in Figure 5d,e. The most important observation from these images is the consistent spherical shape and the evidence of the surface modification. The halo surrounding the pep-AuNPs contrasts the darker core and the lighter background. Figure 5e clearly shows some very defined and perfectly spherical peptide-functionalized gold nanoparticles. The size distribution of the pep-AuNPs is presented in Figure 5f, with the histogram and the normal distribution. A similar tendency to the cit-AuNPs was observed for the pep-AuNPs in terms of dispersity and not perfectly matching the normal distribution. However, in both cases, the distribution of sizes was not perfectly fit with a Gaussian curve, but the average size was representative of the sample, respectively.

**Figure 6:**
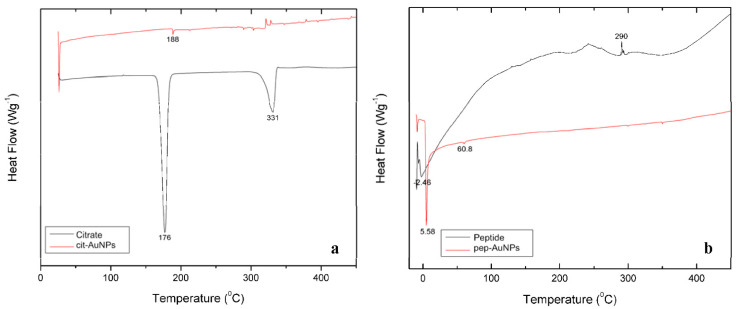
DSC thermograms of (**a**) citrate and cit-AuNPs and (**b**) peptide and pep-AuNPs.

Differential Scanning Calorimetry (DSC) was used to determine if the different ligands (citrate and peptide) attachment to AuNPs increased their thermal stability. The DSC thermogram for trisodium citrate dihydrate showed a peak at 176 °C and another at 331 °C, which corresponds to the two-step dehydration of the salt, as reported in the literature [47] (Figure 6a). However, in the cit-AuNPs, these peaks were shifted to 188 °C and 334 °C. This shift could be associated with the interaction that the inner citrate carboxylate groups generate with the AuNPs surface, which causes the dehydration process to involve more energy. Moreover, two peaks were detected in the cit-AuNPs thermogram at the 330–335 °C range, which may be attributed to the recrystallization of the citrate [48]. Thermograms for the peptide and the pep-AuNPs are presented in Figure 6b. The peptide thermogram shows the presence of two prominent peaks: one at 2.46 °C related to the glass transition of the peptide and the other at 290 °C related to its recrystallization. The thermogram for the pep-AuNPs presented two peaks at 5.58 °C and 60.8 °C. The 5.58 °C peak may be attributed to the glass transition temperature, and the subsequent peak could be attributed to the change in the peptide’s secondary structure. The liquid to glass transition is an inherent feature of the peptide dynamics associated with the breaking/loosening of hydrogen bonds at specific points called defects [48]. The glass transition for the pep-AuNPs was detected at a higher temperature. As demonstrated in the FTIR spectra (Figure 4b), some of the hydrogen interactions of the peptide were replaced with the interactions established with the AuNPs surface, conferring higher stability to it. Moreover, because a small sample could be recuperated after lyophilization, the representative peaks of the thermal shifts were not very intense. They exhibited an uneven distribution of heat flow. However, the other complementary techniques evidence the colloidal stability and the surface of the AuNPs’ interaction with the ligands.

**Figure 7:**
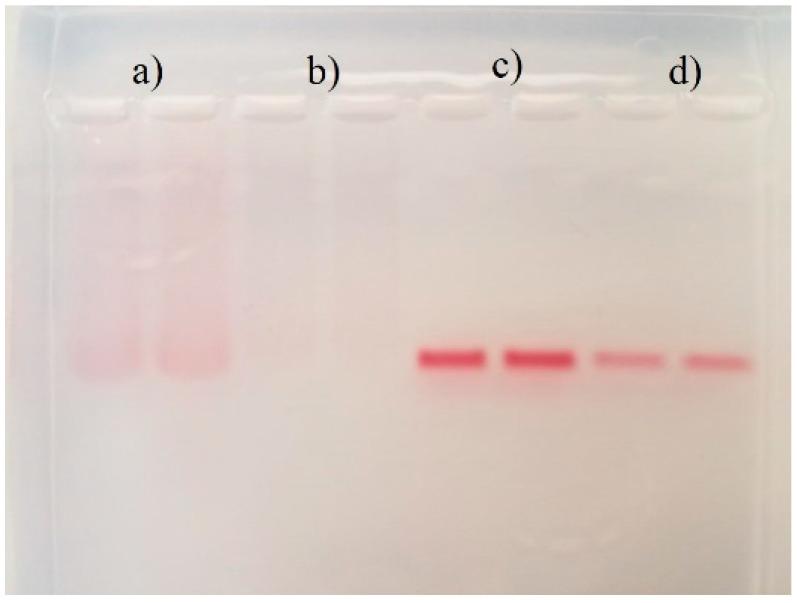
Agarose electrophoresis (1% gel) in TBE 1x running buffer. (**a**) cit-AuNPs, (**b**) washed cit-AuNPs, (**c**) pep-AuNPs, and (**d**) washed pep-AuNPs.

A different approach was employed with a qualitative assessment of the stability of the AuNPs with agarose gel electrophoresis. This technique was used to observe the size dispersity of samples, stability of the particles after washing, and successful modifications of AuNPs (Figure 7). The size distribution of the material was observed during the migration as the samples ran at the same distance as their respective unwashed form. This demonstrates that even after centrifuge runs, AuNPs have the potential to remain unchanging after the stripping of surrounding stabilizing agents, such as citrate, out of the solution. Streaking of the bands in washed and unwashed cit-AuNPs supports the evidence of size dispersity of AuNPs [49]. The aggregation of nanoparticles may cause difficulty in migrating samples, and hence, they stay higher up in the gel [50]. Those that were not aggregated managed to travel further due to their smaller size, and the observed streak was generated. On the other hand, the pep-AuNPs migrated in a very defined and narrow band. This effect can be attributed to the surface charge provided by the peptide itself and the interactions of the anchored peptide with the loading buffer of choice. The loading buffer consisted of a 1:1 ratio of glycerol and SDS 10% solution. This last one can particularly hold onto big organic groups, such as peptides, better than smaller ones, like citrate. This helped aid in the migration in the gel’s travel by amplifying the negative charge of pep-AuNPs. Moreover, the bands corresponding to pep-AuNPs showed significantly lower streaking due to the peptide’s capacity to stabilize and firmly wrap the AuNPs to protect them from clumping up [22]. Short peptides have been shown to dramatically increase the stability of the gold nanoparticles, even providing the possibility of being stored as dry powders without losing their properties after redispersion [51]. Because of the differences in sample migration, gel electrophoresis was a practical and straightforward method to observe and conclude tendencies of size dispersity, aggregation, stability, and proper modifications of nanoparticles in a single test.

**Figure 8:**
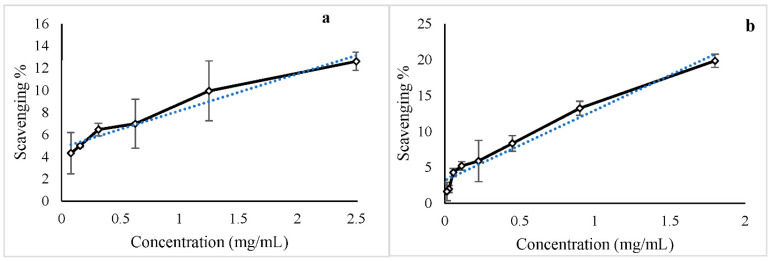
Scavenging effect of different concentrations of gold nanoparticles for the ABTS radical. A linear regression (blue dotted line)was applied, and the IC50 concentration was calculated. (**a**) cit-AuNPs (R^2^ = 0.9544; IC50 = 13.59 mg/mL); (**b**) pep-AuNPs (R^2^ = 0.9649; IC50 = 4.79 mg/mL).

The antioxidant capacity of the cit-AuNPs and pep-AuNPs was evaluated with the ABTS assay. Tendencies shown in Figure 8 demonstrate that the scavenging activity of the AuNPs follows a linear dependency on concentration, which was expected from the dose-dependent nature of the gold nanoparticles as antioxidants [52]. The antioxidant activity was more potent in the pep-AuNPs with an IC50 of 4.79 mg/mL, while the cit-AuNPs were calculated at 13.59 mg/mL. The addition of peptides to stabilize the nanoparticles could also imply an enhanced set of properties for the new materials [52]. In this case, the antioxidant capacity of the modified particle was significantly increased.

**Figure 9:**
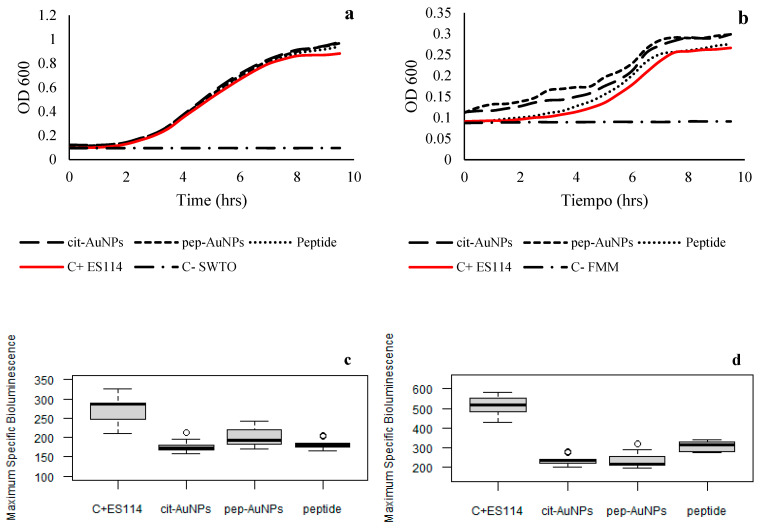
Growth patterns and maximum specific bioluminescence of ES114 exposed to gold nanoparticles in SWTO and FMM media. (**a**) Growth patterns in SWTO when exposed to cit-AuNPs and pep-AuNPs. (**b**) Growth pattern in FMM when exposed to cit-AuNPs and pep-AuNPs. (**c**) Maximum of specific bioluminescence in SWTO when exposed to cit-AuNPs and pep-AuNPs (*p*-values < 0.001). (**d**) The maximum of specific bioluminescence in FMM when exposed to cit-AuNPs and pep-AuNPs (*p*-values < 0.001).

The ecotoxicity of AuNPs was evaluated with a model organism, *Aliivibrio fischeri* ES114. This marine bacterium was used to measure this type of nanomaterials’ impact on marine ecosystems. *Aliivibrio fischeri* is a well-studied microorganism with a very adaptable and versatile metabolism due to its ability to be free-living in the seawater, the adaptability to create biofilms, and its capacity to colonize and undergo a symbiotic relationship with marine animals, such as the bobtail squid, *Euprymna scolopes* [53,54]. The two types of media are used to simulate the different conditions in which the bacteria can be found in the wild, where the minimal media would be comparable to the seawater; thus, the free-living organisms would have similar behaviors, and the complex or rich media could simulate the environment a host could provide for the bacteria. Growth patterns and bioluminescence emission levels are presented in Figure 9. The growth pattern of ES114 in rich media SWTO appeared to not be affected by the presence of the AuNPs, since all the lines in Figure 9a are almost identical and superimposed. However, the bioluminescence was observed to decrease in the exposed groups. This inhibition of bioluminescence is often used to represent the toxicity of the materials evaluated since the bioluminescence emission is related to the aerobic metabolism of the bacteria [55]. The cit-AuNPs and the peptide alone produced significantly lower bioluminescence (Table 2) when compared to the pep-AuNPs. This could suggest that the peptide-stabilized gold nanoparticles are less toxic in this media, which could be related to the increased stability of the pep-AuNPs. Metallic nanoparticles have a few toxicity mechanisms of action, in which the principal is the liberation of the metallic ions to the media, where they can interact with the bacteria [25]. Thus, it was expected that the more stabilized particles would present less inhibition of the bioluminescence. On the other hand, the behavior observed in the minimal media was different. The growth patterns appeared to be affected by the addition of the AuNPs and the peptide. It is known that bacteria placed in minimal media after being successfully grown in complex media will undergo a stressful period [56]. However, the growth patterns may also be affected by the particles’ contribution to the turbidity of the minimal media, which is colorless. Nevertheless, the general growth in the minimal media was prolonged and could not reach a significant absorbance level compared to the rich media growth. The presence of the AuNPs in the minimal media appeared to be highly toxic due to the decreased signal of bioluminescence when compared to the control group. The effect of the peptide seems to be harmful to a certain level, because the bioluminescence is significantly reduced compared to the control but is higher than the measurements of the AuNPs (Table 3). This could suggest that the bacteria are either adapting to the presence of the peptide or are using it as a nutrient in the stressful environment that is the minimal media.

## 4. Conclusions

The presented study demonstrates an effective method to functionalize gold nanoparticles with microalgal peptides. The peptide modification increased the material’s stability and antioxidant capacity and decreased the toxicity of *Aliivibrio fischeri* in rich and minimal media. Moreover, an agarose gel electrophoresis method was optimized to qualitatively observe differences in sizes, aggregation, stability, and evident modifications of AuNPs in scenarios where there is no access to high-end instrumentation, such as the Transmission Electron Microscope. To empower microalgae peptides’ action in pharmaceuticals, food, or cosmetics, they must be able to resist adverse external factors. The use of AuNPs as carriers for microalgae peptides was shown to be an effective strategy to increase peptides protection. Our results represent a model for developing the microalgae-AuNPs systems, which has the potential to be beneficial for a wide range of biological and industrial applications, in addition to catalysis, image enhancement, and sensing.

## Figures and Tables

**Table 1:** Z-average, Intensity Percentage, PDI, and Zeta-potential data of cit-AuNPs and pep-AuNPs.

Sample	Z-Average	First Peak (nm)	Second Peak (nm)	PDI	Zeta-Potential (mV)
cit-AuNPs	22.26	18.94	654.57	0.410	−44.14
pep-AuNPs	27.72	20.97	572.03	0.496	−42.96

Third peak mean values are not available, as due to the small intensity percentages, they could not be integrated.

**Table 2:** Single-factor ANOVA of the maximum specific bioluminescence measurements of the bacteria in SWTO.

ANOVA						
Source of Variation	SS	df	MS	F	*p*-Value	F Crit
Between Groups	53,521.483	3	17,840.49	29.679	2.280 × 10^−9^	2.901
Within Groups	19,235.606	32	601.11			
Total	72,757.089	35				

**Table 3:** Single-factor ANOVA of the maximum specific bioluminescence measurements of the bacteria in FMM.

ANOVA						
Source of Variation	SS	df	MS	F	*p*-Value	F Crit
Between Groups	446,835.939	3	148,945.3	101.281	2.033 × 10^−166^	2.901
Within Groups	47,059.493	32	1470.6			
Total	493,895.432	35				

## Data Availability

The data are available upon request.
